# A force-compensated compliant MEMS-amplifier with electrostatic anti-springs

**DOI:** 10.1038/s41378-023-00557-5

**Published:** 2023-06-29

**Authors:** Philip Schmitt, Martin Hoffmann

**Affiliations:** Microsystems Technology, Ruhr-Universität Bochum, Universitätsstr. 150, 44801 Bochum, Germany

**Keywords:** Electrical and electronic engineering, Sensors

## Abstract

In this paper, an electrostatic compliant mechanical amplifier intended for force-compensated displacement amplification in MEMS sensor applications is described. Usually, mechanical transformers that enhance a small input displacement into a large output displacement generate large forces at the input of the transformer. The microsystem proposed here allows for the reduction and compensation of the input stiffness of the amplifier and any mechanical components connected to it while providing a constant amplification ratio at the same time. The amplifying mechanism features bidirectional electrostatic anti-springs enabling the control of the stiffness by applying a constant DC voltage. The electrode design of the anti-springs and its influence on the force-displacement characteristic, the side instability and the maximal displacement are studied through analytical approaches and supported by FEA and by experiments. Based on the derived models, a compliant electromechanical amplifier is developed, featuring an amplification ratio of 50. For this amplifier the initial input stiffness of 422 N/m could be reduced to 6.8 N/m by applying a voltage of 100 V. As an additional application, we show how the amplifier can be used as a mechanical force sensor with tuneable sensitivity, where the forces at the input are transformed into large output displacements. Through experiments, we show how the sensitivity can be adjusted and increased by a factor of 25 by applying a voltage at the anti-springs.

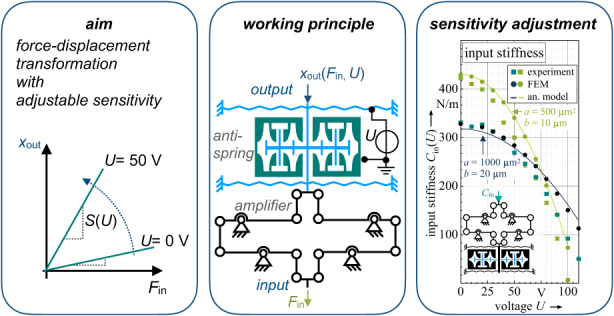

## Introduction

Compliant mechanical displacement amplifiers have been investigated for various applications and can be found in MEMS sensor systems for displacement or force amplification, such as in accelerometers or gyroscopes^1–6^. Mechanical amplifiers are also known from electrostatic^7,8^ or piezoelectric^9,10^ actuators to convert forces into large displacements.

In previous works^11^, we presented the design and modeling of a passive lever-based compliant mechanical amplifier (mechAMP) with an amplification ratio of up to 200. An advantage of compliant amplifiers is that these mechanisms based on flexure hinges do not suffer from wear or backlash. However, compliant hinges generate force reactions in response to imposed displacements, which are also amplified within the mechanism. Additionally, the stiffness of additional springs connected to the output of an amplifying mechanism is increased, resulting in a very large stiffness at the input. Therefore, the resulting stiffness at the amplifier’s entrance strongly depends on the amplification ratio. When aiming for high amplification ratios, the mechanism usually requires extremely high forces at the entrance of the system that are often not compatible with the weak signal to be amplified.

In this paper, we present a force-compensated mechanical amplifier featuring bidirectional electrostatic anti-springs, which allows the amplification of small displacements without feeding back high reaction forces. Furthermore, the proposed amplifier can be used as a mechanical impedance transformer, allowing the input stiffness of the amplifier to be individually set at a given amplification ratio.

Electrostatic stiffness adjustment, as applied here for the mechAMP, is well known for other MEMS applications, especially for MEMS resonators^12–16^. Basically, two methods of electrostatic stiffness adjustment for comb-drive actuators have been investigated thus far. One type focuses on varying the overlapping surface area of parallel plate electrodes in a comb drive by designing finger electrodes of different lengths, as presented in^15–18^. The number of finger electrodes contributing to the force generation varies with the displacement of the actuator. This allows discrete position-dependent force generation. Additionally, a variation of the surface area in the vertical direction that occurs by increasing the thickness of the finger electrodes using greyscale lithography has been introduced^19^.

Another possibility for electrostatic stiffness adjustment consists of the variation of the gap between the finger electrodes to achieve a displacement-dependent electrostatic force. Ye et al.^20^ first presented various electrode shapes that tailor the polynomial electrostatic force-displacement characteristics. An extended study for multiple electrode shapes was presented by Jensen et al.^21^. A detailed review of the methods for mechanical and electrostatic stiffness adjustment is also provided^22^. However, most of the known concepts are suitable for displacements in only one direction and often feature an offset in the force-displacement characteristic.

For the realization of the force compensation of the mechAMP in this work, we pursue the concept of varying electrode gaps for a bidirectional and offset-compensated electrostatic anti-spring.

In the section “System and Design”, the design of the electrostatic anti-spring is studied considering its limitations as well as the integration within the mechAMP. The fabrication process and the experimental model verification are presented in the section “Fabrication and Characterization”, while the section “Conclusion” is a summary of the main results.

## System and design

### General working principle

**Fig. 1:**
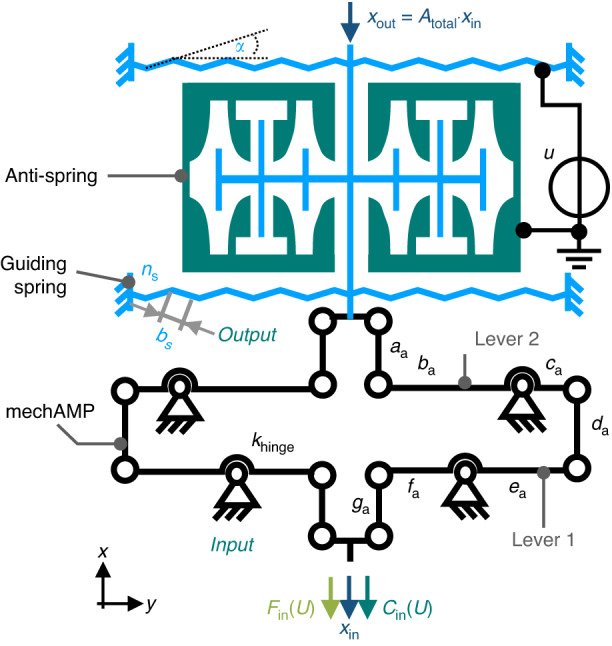
Working principle of the force-compensated mechanical amplifier. The combination of a lever-based compliant amplifier with a bidirectional electrostatic anti-spring allows the reduction and control of the mechanical input stiffness ${C}_{{\rm{in}}}(U)$ of the amplifier. Highly selective triangular guiding springs are used to guarantee high robustness against side-instability for large displacements

As shown in Fig. 1, the compliant amplifier consists of cascaded levers that are serially connected by beams. An electrostatic anti-spring is positioned at the output of the amplifier for force and stiffness compensation.

Based on the serial arrangement of the levers, a small displacement at the system entrance is amplified within two stages. A first amplification is obtained by the first lever and then again by the second lever. A higher cascading with additional levers is possible in principle. In a simplified approximation, the total amplification ratio ${A}_{{\rm{total}}}$ can be seen as the result of the product of the amplification ratios provided by each lever in the serial arrangement:

1 $${A}_{{\rm{total}}}={A}_{1}{A}_{2}\approx \frac{{c}_{{\rm{a}}}}{{b}_{{\rm{a}}}}\frac{{f}_{{\rm{a}}}}{{e}_{{\rm{a}}}}$$

Due to the cascaded arrangement, high amplification ratios can be achieved on a very small footprint. However, the ratio of the input stiffness ${C}_{{\rm{in}}}$ to the output stiffness ${C}_{{\rm{out}}}$ is directly coupled to the amplification ratio by

2 $$\frac{{C}_{{\rm{in}}}}{{C}_{{\rm{out}}}}=\frac{{c}_{{\rm{a}}}^{2}\,{f}_{{\rm{a}}}^{2}}{{b}_{{\rm{a}}}^{2}\,{e}_{{\rm{a}}}^{2}}\approx {A}_{{\rm{total}}}^{2}.$$

High amplification ratios consequently result in high stiffness transformations from one side of the amplifier to the other side. To compensate for the high mechanical input stiffness, electrostatic anti-springs with a negative spring rate are designed and implemented at the output of the amplifier. Integrating the electrostatic anti-springs at the mechAMP’s input instead of the output would compensate the input stiffness as well; however, by implementing the anti-spring at the output, the stiffness conversion of (2) can be used to reduce the number of required anti-springs.

### Design of the electrostatic anti-spring

**Fig. 2:**
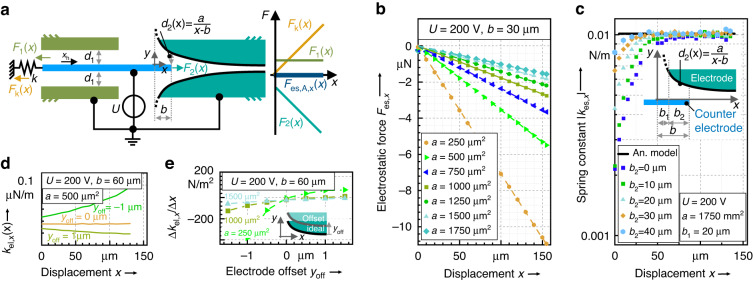
Working principle of a uni-directional electrostatic anti-spring. **a** Shows the setup with the main components and parameters of the electrostatic spring. The curved electrodes generate an offset-affected force ${F}_{2}(x)$ in the x-direction while the parallel-plate actuators on the opposite side generate a constant and offset-compensating counter force ${F}_{1}(x)$. The slope of the resulting proportional force-displacement characteristic can be adjusted by the parameter *a* or by the voltage *U* to compensate the mechanical spring constant ${k}_{{\rm{s}},x}$ of the related mechanical system. **b** Shows results from FEA simulations for the force-displacement characteristics considering various parameters *a* at a constant value for $b=30\,{\rm{\mu m}},\,t=50\,{\rm{\mu m}}$ and $U=200\,{\rm{V}}$. Based on the FEA results in (**b**), Fig. **c** Shows the derived absolute values for the spring rate as a function of the displacement of the counter electrode *x*. **d** Plots the simulated spring rates as a function of the displacement for various offset positions of the curved electrodes. **e** Shows the sensitivity of the spring rate variation as a function of the offset displacement ${{\rm{y}}}_{{\rm{off}}}$ of the curved electrodes for different electrode shapes defined by the parameter *a*. For all simulation results a constant depth of $t=50\,{\rm{\mu m}}$ was assumed

As shown in Fig. 2a, the anti-spring is utilized as a symmetric electrostatic comb-drive actuator with pairs of curved and parallel-plate electrodes. Both types of electrodes generate a linear but negative force-displacement characteristic. While the actuator with curved electrodes provides a linear force-displacement characteristic ${F}_{2}(x)$ with a slight offset, the parallel plate actuator compensates for this offset by providing a constant force ${F}_{1}(x)$ in the opposite *x*-direction. The superposition of both electrostatic forces results in:

3 $${F}_{{\rm{es}},{\rm{A}},x}(x)=-\varepsilon \frac{t}{{d}_{2}(x)}{U}^{2}+\varepsilon \frac{t}{{d}_{1}}{U}^{2}$$

Here, ${d}_{1}$ is the distance of the parallel plate electrodes given by

4 $${d}_{1}=\frac{a}{b}.$$

The function ${d}_{2}(x)$ describes the distance of the curved electrode to the corresponding counter electrode:

5 $${d}_{2}\left(x\right)=\frac{a}{\left(x+b\right)}.$$

Supposing a position of the coordinate system, as shown in Fig. 2a, the function ${d}_{2}(x)$ also defines the shape of the upper curved electrode with its proportional factor $a$ given in [m^2^] and the shifting parameter $b$ in [m].

After inserting (4) and (5) into (3) and dividing by the displacement *x*, the negative spring constant of the electrostatic spring yields:

6 $${k}_{{\rm{es}},{\rm{A}},{\rm{x}}}=\frac{{F}_{{\rm{es}},x}(x)}{x}=-\frac{\varepsilon \,t}{a}{U}^{2}$$

As seen from (6), the spring rate mainly depends on the applied voltage $U$ and on the shape of the curved electrodes described by the parameter $a$. The parameter $b$ is eliminated by the additional parallel plate actuator according to (4). By neglecting the shifting parameter $b$ or choosing a small value for $b$ in the electrode design, the additional compensating parallel plate actuator would become superfluous, but simultaneously, the dimensions of the electrode and thus its footprint would be notably increased, as seen in (5) $(\mathrm{with}\mathop{{\rm{lim}}}\nolimits_{b\to 0}{d}_{2}\left(x\right)=\infty)$.

To verify the model provided by (3) and (6), a finite element analysis (FEA) of different counter-spring designs was carried out in *COMSOL Multiphysics*. The results of the analytic model and of the FEA are compared in Fig. 2b for different electrode shapes defined by the parameter *a*. Although Fig. 2b shows good compliance between the models, small deviations can be observed for displacements below 50 µm.

At small displacements around the initial *x*-position, neglected stray fields limit the validity of the analytic model. To reduce such parasitic effects, the shifting parameter $b$ can be split into two factors ${b}_{1}$ and ${b}_{2}$, as indicated in Fig. 2c. While ${b}_{1}$ defines the position at the starting point of the curved electrode regarding the origin of the imposed coordinate system, the parameter ${b}_{2}$ describes the initial overlap of the curved electrode and the counter electrode. As shown in Fig. 2c, the consistency of ${k}_{{\rm{es}},{\rm{x}}}(x)$ can be significantly improved by increasing the initial overlap, which reduces the influence of the stray field.

Within the FEA, the impact of a small offset displacement ${y}_{{\rm{off}}}$ of the curved electrode in the *y*-direction in reference to its ideal position (5) is analysed. Here, the curved electrode is shifted to determine the impact of small *y*-displacements due to fabrication tolerances.

Figure 2d reveals that the electrostatic spring rate reacts very sensitively to a vertical shift of the electrodes. For small offsets such as 1 µm, the electrostatic anti-spring loses its property to provide a constant spring rate and instead shows a rather nonlinear force-displacement characteristic.

The sensitivity of the constancy of the spring rate to the offset-displacement is analysed in Fig. 2e. The diagram shows the variation of the spring constant $\Delta {k}_{{\rm{es}},{\rm{x}}}(x)/(\Delta x)$ to an imposed displacement of the counter electrode in the *x*-direction as a function of the electrode offset position ${y}_{{\rm{off}}}$ for different electrode designs. Figure 2e shows that the sensitivity of the spring rate to the offset displacement strongly decreases for electrodes with rising values of *a*.

Therefore, large values for *a* should be selected for the electrode design when aiming for high immunity to fabrication tolerances.

**Fig. 3:**
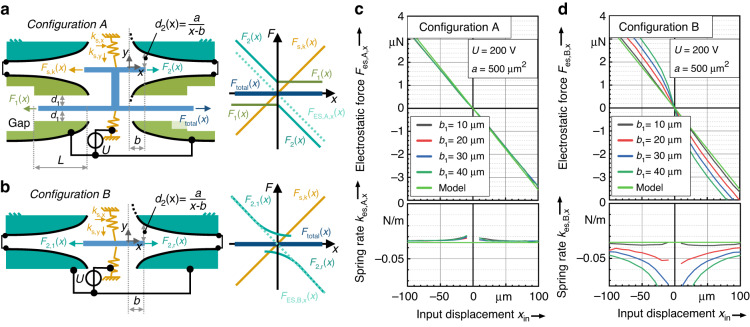
Design of the bidirectional anti-spring with offset compensation. **a** Shows Configuration A consisting of two parallel plate actuators and two curved electrode actuators. The initial overlap *b*_2_ is set to zero. A gap at the parallel plate actuators limits its impact to one direction of displacement, respectively. Stray fields are neglected. In Configuration B. **b** the offset compensation is achieved through a symmetrical arrangement of two curved actuators, where the stray fields compensate the force offset. The diagram in Fig. **c** Shows the FEA results for the force-displacement characteristics and the resulting spring rate for Configuration A considering various electrode designs defined by different values of *b*_1_. The FEA results match the results obtained by the model in (6). **d** Shows the FEA results obtained for Configuration B considering various electrode designs defined by *b*_1_. Here, *b*_2_ is zero, too. The comparison to the model given by (7) shows that Configuration B only shows a linear anti-spring characteristic for small values of *b*_1_. For the simulations, an electrode depth of $t=50\,{\rm{\mu m}}$ was assumed

The proposed configuration in Fig. 2a also allows a bidirectional design of the actuator, shown in Fig. 3a as *Configuration A*. Here, the combination of curved electrodes and parallel plate actuators is set up in a mirrored arrangement. To limit the influence of the parallel plate actuators to one direction of displacement, a gap is introduced at the end of each actuator. For *Configuration A*, we assume that the electrostatic force ${F}_{2}(x)$ of the curved electrodes only applies if the curved and counter electrodes overlap. However, even outside the overlapping area, stray fields generate forces that have an impact on the counter electrodes.

Figure 3b shows *Configuration B*, where only a mirrored arrangement of curved electrodes is used, and the parallel plate actuators are omitted. Due to the symmetric setup, the force offset within the force-displacement characteristic of the curved actuator is compensated by a counter force generated by the stray fields of the opposed actuator. For *Configuration B*, the overlap should be ${b}_{2}=0$. If the electrodes have an initial overlap ${b}_{2} > 0$, the displacement-dependent electrostatic force and thus the value of the electrostatic spring constant doubles, resulting in:

7 $${F}_{{\rm{es}},{\rm{B}},x}\left(x\right)=\left\{\begin{array}{c}-\varepsilon \frac{t}{{d}_{2}\left(x\right)}{U}^{2}+\varepsilon \frac{t}{{d}_{2}\left(-x\right)}{U}^{2}=-2\frac{\varepsilon \,t\,x}{a}{U}^{2},\,x \, < \, {b}_{2}\\ -\varepsilon \frac{t}{{d}_{2}\left(x\right)}{U}^{2}+\varepsilon \frac{t}{{d}_{2}\left(0\right)}{U}^{2}=-\frac{\varepsilon \,t\,x}{a}{U}^{2},\,x\ge {b}_{2}\end{array}\right.$$

The force displacement characteristic of *Configuration B* shows a linear and proportional behavior as long as the overlapping state does not change from overlapping to not overlapping within the travel range of the displaced counter electrode.

Figure 3c shows the plotted force-displacement characteristic in comparison to the results of an FEA for *Configuration A*. The electrostatic spring rate yields a constant behavior after overcoming the effects at the initial *x*-position of the counter electrode, which is almost independent of the varying design parameter ${b}_{1}$.

In contrast, the force-displacement characteristic of *Configuration B* indicates a strong dependence on the electrode design. Figure 3d shows the results of an FEA for a varying parameter ${b}_{1}$. The diagram reveals that the theoretical values given by (7) are confirmed through FEA only if the parameter ${b}_{1}$ is sufficiently small.

### Side instability and maximal displacement

As known from comb-drive actuators^23^, the maximum achievable displacement is usually restricted by side instability. This limit is caused by a pull-in of the electrodes at a displacement ${x}_{{{\max }}}$, which is normally defined as the point where the electrostatic stiffness of the actuator exceeds the stiffness of the mechanical guiding springs in the off-axial direction.

**Fig. 4:**
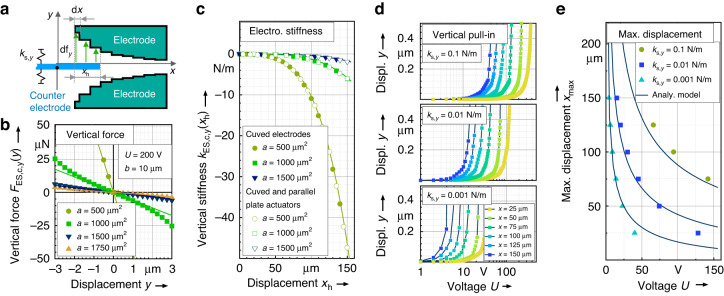
Vertical force and side-instability **a** Illustrates the integrational concept of the vertical force determination. **b** Provides a model verification of the vertical force model given in (10) by comparison to the FEA results. The vertical force on a counter electrode positioned between two curved electrodes and displaced by ${x}_{{\rm{h}}}=150$ ${\rm{\mu m}}$ was determined as a function of the vertical displacement in the y-direction. Lines indicate the analytical results, while dots show the FEA results. **c** Compares the analytical and FEA results for the vertical stiffness of a counter electrode between two curved electrodes as a function of the displacement ${x}_{{\rm{h}}}$. Simulations were carried out for pairs of counter electrodes and curved electrodes, only (filled symbols) and for curved electrodes with additional parallel plate actuators (unfilled) required for configuration A. **d** Shows the FEA results for the pull-in behavior of the curved electrode actuator. The diagrams indicate the vertical displacement of a counter electrode between a pair of curved electrodes as a function of the applied voltage for various x-displacements of the counter electrode and thus of various overlapping areas. The three diagrams refer to three different stiffness values of the mechanical guiding mechanism, as indicated in (**a**). Based on the asymptotic pull-in voltages given in (**d**), (**e**) Shows the maximal displacement ${x}_{{{\max }}}$ of the anti-spring as a function of the applied voltage compared to the results obtained by (14). For the simulations considered in (**c**) the values of $t=50\,{\rm{\mu m}}$, $b=20\,{\rm{\mu m}}$ and $U=200\,{\rm{V}}$ were used while for Figs. (**d**) and (**e**) the actuator was designed with $t=50\,{\rm{\mu m}}$, $a=1750\,{{\rm{\mu m}}}^{2}$ and $b=20\,{\rm{\mu m}}$

For the calculation of the electrostatic stiffness in the off-axial direction (*y*-direction), the corresponding electrostatic force ${F}_{{\rm{ES}},{\rm{c}},y}({x}_{{\rm{h}}},y)$ of the curved actuator can be approximated by an integration of the electrostatic vertical force of overlapping electrodes supposing that the infinitesimal small parallel plate actuators are arranged, as illustrated in Fig. 4a. For a number of *n* pairs consisting of one curved electrode and one counter electrode, the vertical force ${F}_{{\rm{ES}},{\rm{c}},y}({x}_{{\rm{h}}},y)$ results in:

8 $${F}_{{\rm{ES}},{\rm{c}},y}({x}_{{\rm{h}}},y)=\frac{1}{2}\,n\,\varepsilon \,t{U}^{2}{\int }_{0}^{{x}_{{\rm{h}}}}\frac{1}{{\left(\frac{a}{b+x}-y\right)}^{2}}{\rm{d}}x.$$

The electrostatic stiffness ${k}_{{\rm{es}},{\rm{c}},y}\left({x}_{{\rm{h}}},y\right)$ as a function of the displacement ${x}_{{\rm{h}}}$ and $y$ for a curved electrostatic actuator is then given by:

9 $${k}_{{\rm{ES}},{\rm{c}},y}\left({x}_{{\rm{h}}},y\right)=\frac{{\rm{d}}}{{\rm{d}}y}\left({F}_{{\rm{es}},{\rm{c}},y}\left({x}_{{\rm{h}}},y\right)-{F}_{{\rm{es}},{\rm{c}},y}\left({x}_{{\rm{h}}},-y\right)\right)$$

Solving (9) and (8) for the proposed geometry defined in (5) results in a large term that can be simplified by a linear Taylor series at the point $y=0$, resulting in:

10 $$T\left({k}_{{\rm{ES}},{\rm{c}},y}\left({x}_{{\rm{h}}},y\right),y=0\right)\approx \frac{\varepsilon \,n\,t\,{U}^{2}\left(4\,{b}^{3}x+6\,{b}^{2}{x}^{2}+4\,b\,{x}^{3}+{x}^{4}\right)}{2\,{{\rm{a}}}^{3}}$$

To verify the last approximation, an FEA was carried out to simulate ${F}_{{\rm{ES}},{\rm{c}},y}\left({x}_{{\rm{h}}},y\right)$ depending on the vertical *y*-displacement. The results are plotted in Fig. 4b, showing good accordance for small deviations of *y*.

In addition to the curved electrodes, the parallel plate actuators that are required for the offset compensation also contribute to the vertical electrostatic stiffness. This vertical stiffness ${k}_{{\rm{ES}},{\rm{pp}},y}\left({x}_{{\rm{h}}},y\right)$ for parallel plate actuators is known from Reference. ^23^ or Reference. ^24^ and can be determined by solving (9) considering a constant electrode gap of ${d}_{1}$, which yields:

11 $${k}_{{\rm{ES}},{\rm{pp}},y}\left({x}_{{\rm{h}}},y\right)=\frac{2\,t\,\varepsilon \,n\,(2L-{x}_{{\rm{h}}})\,{U}^{2}}{{d}_{1}^{3}}$$

Here, $L$ is the initial overlap of a single parallel plate actuator, as indicated in Fig. 3a. The influence of the parallel plate actuator on the overall vertical anti-spring stiffness is quite restricted, as shown in Fig. 4c. The diagram shows the various FEA simulation results for the vertical stiffness considering both cases: the stiffness of a system comprising the parallel plate actuator and the curved actuator as well as the stiffness of the curved electrodes only. The influence of the parallel plate actuator can be neglected for simplification if $b$ is much smaller than $L$. For example, defining a deviation of 1% with

12 $$\frac{{k}_{{\rm{ES}},{\rm{pp}},y}\left({x}_{{\rm{h}}},y\right)}{{k}_{{\rm{ES}},{\rm{c}},y}\left({x}_{{\rm{h}}},y\right)}\le 1 \%$$

leads to the design rule $b\le L/6$, which is assumed for all further investigations.

The largest possible displacement ${x}_{{{\max }}}$ of the anti-spring is achieved when the value of the mechanical spring constant ${k}_{{\rm{s}},y}$ in the off-axial direction is exceeded by the electrostatic spring constant ${k}_{{\rm{ES}},{\rm{c}},y}$ of the curved electrode, neglecting the influence of the parallel plate actuator:

13 $${k}_{{\rm{es}},{\rm{c}},y}\le {k}_{{\rm{s}},y}$$

By inserting (10) in the last inequation and solving for *x*, a possible solution for the maximal displacement yields:

14 $${x}_{{{\max }}}\le -b+\frac{{\left(\varepsilon nt{U}^{2}\left(2{a}^{3}{k}_{{\rm{s}},y}+{b}^{4}{nt\varepsilon U}^{2}\right)\right)}^{\frac{1}{4}}}{U\,\sqrt{n\,t\,\varepsilon \,}}$$

The last inequation is verified by a series of FEA simulations considering a pair of curved electrodes and a spring-based counter electrode. A simulation of the pull-in was provoked for various *x*-displacements of the counter electrode by increasing the voltage, as shown in Fig. 4d. The simulations were repeated for different ${k}_{{\rm{s}},y}$ of the guiding spring. From the resulting asymptotic pull-in voltages given in Fig. 4d, the maximal displacement ${x}_{{{\max }}}$ at a given voltage was determined and is plotted in Fig. 4e. The results obtained from the FEA are compared to the model given in (14), revealing a sufficient accordance allowing the simplified model to be used.

### Implementation of the electrostatic anti-spring at the mechanical amplifier

The electrostatic anti-spring is mounted at the output element of the mechAMP to compensate for the high input stiffness. In a purely mechanical configuration, the input stiffness ${C}_{{\rm{in}}}$ depends on the rotational stiffness of the flexure hinges ${k}_{{\rm{h}}}$ as well as on the segment lengths of the levers, whereby only the lever segments ${b}_{{\rm{a}}},{c}_{{\rm{a}}}$ and ${e}_{{\rm{a}}}$ have a significant influence on the stiffness^11^. In addition, the input stiffness ${C}_{{\rm{in}}}$ also comprises the amplified stiffness ${k}_{{\rm{s}},x}$ of any guiding spring positioned at the output of the mechAMP. Based on ref. ^11^ and on the model of the negative spring constant provided in (6), the input stiffness of a two-stage amplifier can be approximated by

15 $${C}_{{\rm{in}}}\left(U\right)=\frac{{F}_{{\rm{in}},y}}{{y}_{{\rm{in}}}}=6\,{k}_{{\rm{h}}}\,\frac{{e}_{{\rm{a}}}^{2}+{c}_{{\rm{a}}}^{2}}{{b}_{{\rm{a}}}^{2}{e}_{{\rm{a}}}^{2}}\,[{\rm{rad}}]+{A}_{{\rm{total}}}^{2}\left({k}_{{\rm{s}},x}-\frac{\varepsilon \,t\,n}{a}{U}^{2}\right)$$

Here, ${k}_{{\rm{h}}}$ is given in [N ∙ m/rad]. With the electrostatic actuator positioned at the amplifier’s output, the electrostatic force and thus the negative electrostatic spring constant are amplified by the mechAMP. Consequently, the higher the amplification ratio ${A}_{{\rm{total}}}$ is, the higher the impact of the anti-spring.

The last equation also shows that the force-compensated amplifier can be used as a tuneable force sensor. Here, a force at the input of the mechAMP is converted into a displacement at the output, and the sensitivity of the force sensor is tuneable by the voltage applied at the anti-spring:

16 $${y}_{{\rm{out}}}({F}_{{\rm{in}},y},U)={A}_{{\rm{total}}}\frac{{F}_{{\rm{in}},y}}{{C}_{{\rm{in}}}(U)}$$

## Fabrication and characterization

### System design

For the design of the demonstrators, a mechanical amplifier with amplification ratios of$\,{A}_{{\rm{total}}}=25$ and ${A}_{{\rm{total}}}=50$ were chosen featuring flexure hinges with a width of 2 µm that results in a nominal stiffness ${k}_{{\rm{h}}}$ of 196 µN m/rad. The relevant dimensions of the lever segments and the dimensions of the electrostatic anti-spring are summarized in Table 1.

**Table 1:** Design parameters of the demonstrators

parameter	V1	V2	V3	unit
$a$	500	1000	1000	µm^2^
$b$	10	20	20	µm
${k}_{{\rm{h}}}$	196	196	196	µN m/rad
${k}_{{\rm{s}},{\rm{x}}}$	80.9	40.45	40.45	mN/m
${k}_{{\rm{s}},{\rm{y}}}$	7.67	3.83	3.83	N/m
$b$	79	79	79	µm
$c$	1586	1586	1586	µm
$d$	475	475	475	µm
$e$	1190	1190	740	µm
$t$	20	20	925	µm
${A}_{{\rm{total}}}$	50	50	25	

**Fig. 5:**
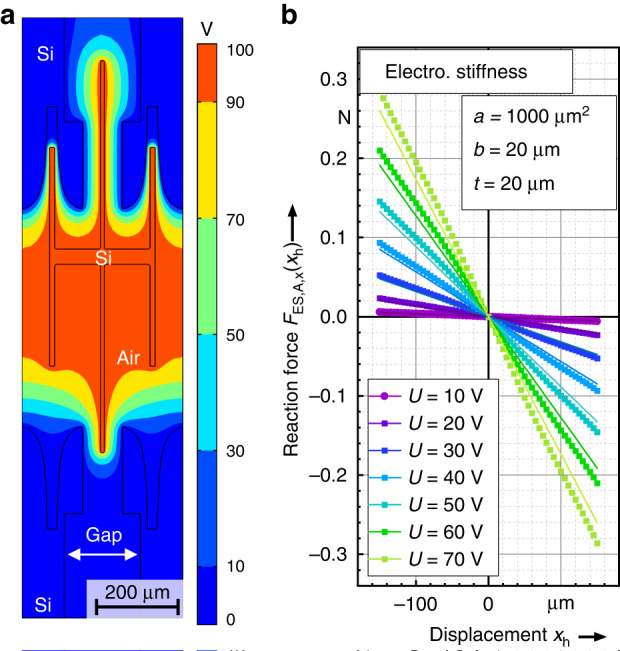
Design and simulation results of the electrostatic anti-spring with *ɑ* = 1000 μm^2^ and *b* =20 μm **a** Shows an exemplary setup for an FEA simulation of the anti-spring showing the potential distribution for a displaced electrostatic anti-spring at a displacement of 120 µm. In (**b**) the force-displacement characteristic of the referred anti-spring is shown for various voltages and compared to the results of the analytic model (6)

In total, 40 pairs of electrodes of *Configuration A* are mounted at the output of the mechAMP for each direction. Figure 5 shows the respective simulation setup for two bidirectional anti-springs as well as the simulated and calculated force-displacement characteristics at various voltages.

Highly selective guiding springs are mandatory for a large translational displacement. On the one hand, the spring provides a high stiffness ${k}_{{\rm{s}},y}$ in the off-axial direction to prevent lateral pull-in. On the other hand, it should exhibit a very low value for the stiffness ${k}_{{\rm{s}},x}$ in the axial guiding direction (*x*-direction). For this reason, we use triangular springs in a *clamped-clamped* configuration, as shown in Fig. 1. By designing springs with a low inclination angle $\alpha$ and a large number of segments ${n}_{{\rm{s}}}$, a high selectivity of the spring rates in different directions can be achieved; details are given in ref. ^25^.

Here, the electrostatic anti-springs are guided by triangular springs featuring ${n}_{{\rm{s}}}$ = 10 segments with an inclination of $\alpha$ = 45° and a segment length of ${b}_{{\rm{s}}}=195 \,{\upmu }{\rm{m}}$. The spring constant ${k}_{{\rm{s}},x}$ of the guiding system was determined through FEA to be 0.01 N/m in the axial direction, while the spring rate in the vertical direction ${k}_{{\rm{s}},y}$ amounts to 0.96 N/m. The selectivity, and thus the ratio of ${k}_{{\rm{s}},y}/{k}_{{\rm{s}},x}$, yields a value of 96.

### Fabrication

**Fig. 6:**
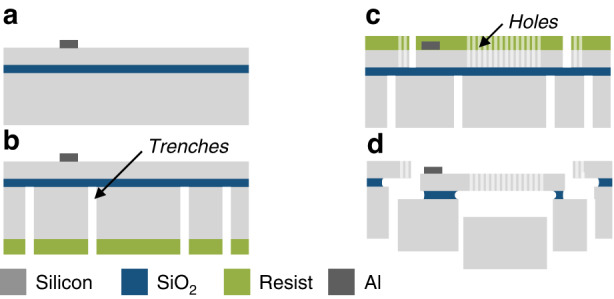
Fabrication process based on SOI-technology

The force-compensated mechanical amplifiers are realized on a (100)-oriented 100 mm SOI substrate featuring a 20 µm device layer. The fabrication process, based on a concept known from refs. ^26,27^, is shown in Fig. 6. First, aluminum is deposited and patterned by wet chemical etching (6a), forming bond electrodes. To release the chips from the substrate without dicing and for the removal of the handle layer in the area of the electrostatic actuators, the 300 µm thick handle layer was patterned by deep reactive ion etching (6b). The mechanical structures of the amplifier were then patterned and etched into the device layer (6c) using a low scallop, low undercut SOI deep reactive ion etching process. Within the design, the device layer also features trenches surrounding the chip boundaries. To ensure proper and complete disassembly of the chips from the wafer, the device layer also includes multiple holes enabling the HF vapor to finally release the mechanical structures (6d).

**Fig. 7:**
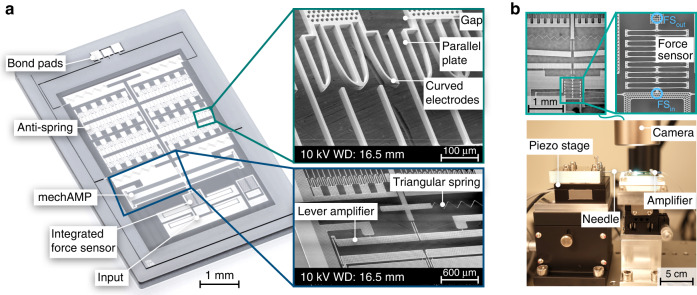
Fabricated demonstrator and experimental setup. **a** Shows a photo stacked image of a fabricated demonstrator with a detailed view (SEM images) of the anti-spring and the mechanical lever amplifier. **b** Shows the measurement setup mainly comprising a microscope camera and a piezoelectric stage for external manipulation of the MEMS using a needle. The microsystems feature integrated force sensors for visual determination of the applied forces by measuring the elongation of the spring

Figure 7a shows a photo stacked image of the fabricated system and a detailed view (SEM) of the curved electrodes and the mechanical amplifier. For characterization, an integrated force sensor is implemented at the entrance of the system.

### Electromechanical characterization

Figure 7b shows the experimental setup for the characterization. The setup comprises a microscope camera, a controllable piezoelectric stage with a fixed tungsten needle and a voltage source that is connected to the electrodes of the anti-springs through a 1 MΩ resistor for current limitation. For characterization, the microsystems also include integrated force sensors, realized as linear springs with a known and calibrated spring constant. The integrated force sensor is calibrated in a weighting cell to determine its linear spring constant ${k}_{{\rm{FS}}}$ (cf. ^25^).

The tungsten needle is used to displace the amplifier externally at the input of the amplifier using the piezo stage. The introduced force ${F}_{{\rm{in}}}={k}_{{\rm{FS}}}\,\Delta {x}_{{\rm{FS}}}$ is measured visually by tracking the spring deflection $\Delta {x}_{{\rm{FS}}}$ of the integrated force sensor.

**Fig. 8:**
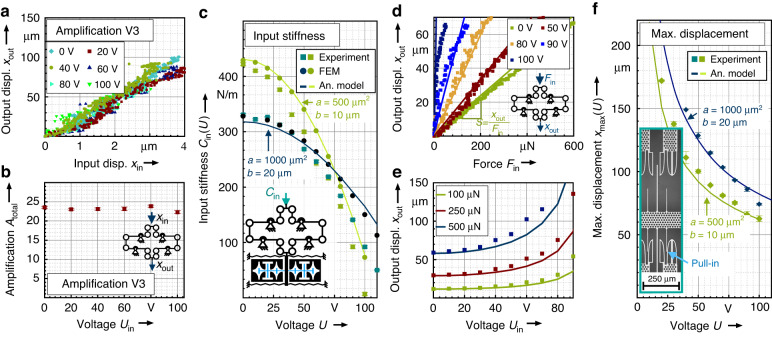
Measurement results. **a** Shows the amplification characteristic based on a mechAMP of Version 3. Here, the output displacement is measured as a function of the imposed input displacement for various voltages applied at the anti-spring. **b** Indicates the measured amplification ratio as a function of the applied voltage. **c** Provides the measured input stiffness ${C}_{{\rm{in}}}(U)$ of the two demonstrator chips (V1,V2) as a function of the applied voltage at the anti-spring. The measurement results are shown in comparison to the results obtained by the analytical model given in (15) and the FEA simulations. The measurement results show the average values of three experiments. **d** Shows the measured output displacement of the amplifier as a function of the force at the input of the amplifier for three different voltages applied to the anti-springs (values shown for Version 1). The diagram also shows the results obtained by the model given by (16). Based on the same model, (**e**) gives the output displacement as a function of the voltage for constant values of the applying input force. **f** Presents the measured maximal output displacement of the amplifier which is limited by side-instability of the electrostatic anti-springs. The measurement results are shown in comparison to the results obtained by (14). The displacement is limited by the side-instability of the electrostatic anti-spring. Each plotted dot shows the average result of three measurements, error bars indicate the standard deviation

Within the characterization, we first analysed the amplification ratio for various applied voltages. As we expect, the anti-spring should not influence the amplification ratio. Based on the visual measuring concept where the input and output displacements are tracked by a microscope camera, a microsystem of version 3 with a low amplification ratio of a nominal value of 25 is used for this experiment. Figure 8a shows the output displacement ${x}_{{\rm{out}}}$ as a function of the imposed input displacement ${x}_{{\rm{in}}}$ for different voltages. The raw data shown in the diagram indicate linear characteristics for each measurement. The noisy character of the signal is due to the resolution of the microscope camera considering the visual tracking of the input displacement in the range of 4 µm only.

Based on the measurements in Fig. 8a, linear fits were determined to identify the amplification ratios ${A}_{{\rm{total}}}$ as a function of the applied voltage. For each voltage, three measurements were performed to find a combined fit function. The slope of the linear fit corresponds to the amplification ratio ${A}_{{\rm{total}}}$. As indicated in Fig. 8b, the amplification ratio is not significantly linked to the applied voltage.

To determine the input stiffness ${C}_{{\rm{in}}}$ of the mechAMP, the force-displacement characteristics of the amplifier input element are measured as a function of the applied voltage of the anti-spring for input displacements of up to 2 µm. The slope of the linear fit of the experimental data finally provides the input stiffness ${C}_{{\rm{in}}}$.

Figure 8c shows the measured results for ${C}_{{\rm{in}}}$ compared to the results of an FEA and the analytic model given by (15). For version 1 ($a=500\,{\rm{\mu }}{{\rm{m}}}^{2}$), the initial input stiffness at 0 V was found to be 422 N/m. At 100 V, the value decreases to 6.8 N/m, approximately 1.6% of the initial value at 0 V. For Version 2 (with $a=1000\,{\rm{\mu }}{{\rm{m}}}^{2}$), the initial measured value of 330.6 N/m decreases to 50.2 N/m at 110 V corresponding to 15%. As expected, the anti-spring with the smaller parameter *a* shows a higher spring rate sensitivity ${C}_{{\rm{in}}}(U)$ with respect to the applied voltage $U$. The measurement results follow the theoretical values given by the analytic model (15) and FEA simulations. The deviation of the absolute values is mainly attributed to fabrication tolerances at the flexure hinges and the guiding springs.

In the theoretical section, according to Eq. (16), we discussed that the mechanical amplifier can also be considered a tuneable force sensor, where the output displacement ${y}_{{\rm{out}}}({F}_{{\rm{in}},y},U)$ depends on the applied input force as well as on the applied voltage.

Considering the mechanical amplifier as a tuneable force sensor, the diagram in Fig. 8d shows the output displacement of the mechAMP (version 1) as a function of the input force for different voltages. The diagram demonstrates the tuning of the sensitivity by the anti-spring. Compared to the results of the model given in (16), the experimental values show a reasonable agreement. At 50 V, the sensitivity of the output displacement as a function of the input force ${y}_{{\rm{out}}}/{F}_{{\rm{in}},y}$ could be increased by a factor of 1.4, reaching 0.17 µm/µN, compared to 0.118 µm/µN at 0 V. At 100 V, the sensitivity is increased by a factor of 25, reaching 2.97 µm/µN, considering a linear fit of the diagram in the interval from 0 to 15 µm.

Figure 8d indicates that the higher the voltage is, the more nonlinear the force-displacement characteristic becomes. This is due to the nonlinear force-displacement characteristic of the triangular guiding springs. The larger the force compensation of the anti-spring, the more dominant the triangular spring becomes. For purely linear guiding springs, a linear characteristic curve is expected.

Figure 8e shows the output displacement of the amplifier ${y}_{{\rm{out}}}({F}_{{\rm{in}},y},U)$ as a function of the applied voltage for constant force values. The diagram shows that for a given input force, the range of the output displacement can be doubled by applying a voltage of approximately 80 V. Considering force measurement applications where the output displacement is used as a force indicator, the diagram shows that the measurement range of the applying force can be easily adjusted by the applied voltage.

As discussed in the theoretical section, the output displacement of the mechanical amplifier is limited by side instability. In Fig. 8f, the maximum output displacement is studied experimentally. By increasing the voltage iteratively at the anti-spring, the output of the mechAMP is displaced up to the lateral pull-in position. Figure 8f summarizes the measured maximal displacement ${x}_{{{\max }}}$ as well as the corresponding results of the analytic model given by (14). The measurement shows good correspondence to the theoretical results. For the demonstrator of version 1, even at 100 V, a maximal output displacement of 62.6 ± 2.3 µm is achievable. For Version 2, the maximal output displacement at 100 V amounts to 74.2 ± 0.8 µm.

## Conclusion

The design, modeling and characterization of an electrostatic compliant micromechanical amplifier is presented in this paper.

The mechanism features 40 bidirectional actuators that allow adjustment of the input stiffness of the mechanical amplifier by applying a DC voltage. It can be shown through experiment that due to this anti-spring, the intrinsic and external stiffness caused by the required components, such as flexure hinges or guiding springs, could be drastically reduced from 422 N/m to 6.8 N/m by applying a voltage of 100 V.

The influence of the design parameters was analysed regarding the stiffness-to-voltage relation of the force-displacement characteristic, the side-instability and thus the maximal displacement of the anti-spring. We could show that increasing the Factor *a* and decreasing the Factor *b* allows large deflections of the anti-spring before pull-in. However, a steep slope of the force-displacement characteristic of the anti-spring is achieved by choosing a small value of *a*. The analytical approach was also confirmed through FEA simulations and experiments. Here, we have shown that the output displacements of over 60 µm are still possible at the maximum voltage of 100 V.

As a potential application, the mechanical amplifier can also be used as a tuneable force sensor transforming an input force into a displacement signal. We were able to show that the sensitivity of the displacement-to-force relation could be tuned by a factor of 25. Additionally, in inertial sensors such as in MEMS accelerometers or gyroscopes, the proposed microsystem can be used to amplify and adjust the sensitivity of the respective sensor.
